# Antiviral activity of four types of bioflavonoid against dengue virus type-2

**DOI:** 10.1186/1743-422X-8-560

**Published:** 2011-12-28

**Authors:** Keivan Zandi, Boon-Teong Teoh, Sing-Sin Sam, Pooi-Fong Wong, Mohd Rais Mustafa, Sazaly AbuBakar

**Affiliations:** 1 Tropical Infectious Disease Research and Education Center (TIDREC), Department of Medical Microbiology, Faculty of Medicine, University of Malaya, Kuala Lumpur, Malaysia; 2 Department of Pharmacology, Faculty of Medicine, University of Malaya, Kuala Lumpur, Malaysia

**Keywords:** Antiviral, Dengue virus, Flavonoid, Quercetin, Naringin, Daidzein, Hesperetin

## Abstract

**Background:**

Dengue is a major mosquito-borne disease currently with no effective antiviral or vaccine available. Effort to find antivirals for it has focused on bioflavonoids, a plant-derived polyphenolic compounds with many potential health benefits. In the present study, antiviral activity of four types of bioflavonoid against dengue virus type -2 (DENV-2) in Vero cell was evaluated. Anti-dengue activity of these compounds was determined at different stages of DENV-2 infection and replication cycle. DENV replication was measured by Foci Forming Unit Reduction Assay (FFURA) and quantitative RT-PCR. Selectivity Index value (SI) was determined as the ratio of cytotoxic concentration 50 (CC_50_) to inhibitory concentration 50 (IC_50_) for each compound.

**Results:**

The half maximal inhibitory concentration (IC_50_) of quercetin against dengue virus was 35.7 μg mL^-1 ^when it was used after virus adsorption to the cells. The IC_50 _decreased to 28.9 μg mL^-1 ^when the cells were treated continuously for 5 h before virus infection and up to 4 days post-infection. The SI values for quercetin were 7.07 and 8.74 μg mL^-1^, respectively, the highest compared to all bioflavonoids studied. Naringin only exhibited anti-adsorption effects against DENV-2 with IC_50 _= 168.2 μg mL^-1 ^and its related SI was 1.3. Daidzein showed a weak anti-dengue activity with IC_50 _= 142.6 μg mL^-1 ^when the DENV-2 infected cells were treated after virus adsorption. The SI value for this compound was 1.03. Hesperetin did not exhibit any antiviral activity against DENV-2. The findings obtained from Foci Forming Unit Reduction Assay (FFURA) were corroborated by findings of the qRT-PCR assays. Quercetin and daidzein (50 μg mL^-1^) reduced DENV-2 RNA levels by 67% and 25%, respectively. There was no significant inhibition of DENV-2 RNA levels with naringin and hesperetin.

**Conclusion:**

Results from the study suggest that only quercetin demonstrated significant anti-DENV-2 inhibitory activities. Other bioflavonoids, including daidzein, naringin and hesperetin showed minimal to no significant inhibition of DENV-2 virus replication. These findings, together with those previously reported suggest that select group of bioflavonoids including quercetin and fisetin, exhibited significant inhibitory activities against dengue virus. This group of flavonoids, flavonol, could be investigated further to discover the common mechanisms of inhibition of dengue virus replication.

## Background

Dengue virus (DENV) is a member of the genus flavivirus of the *Flaviviridae *family. It is a significant human pathogen which causes a wide spectrum of clinical illnesses ranging from a silent or mild febrile infection, self-limited dengue fever (DF) to the severe dengue hemorrhagic fever (DHF) and dengue shock syndrome (DSS). There are four dengue virus genotypes, DENV-1, DENV-2, DENV-3 and DENV-4 which are transmitted to humans mainly by two species of mosquitoes, *Aedes agypti *and *Aedes Albopictus *[1]. All four DENV can cause dengue. To date there are no effective vaccine or antiviral treatment for dengue. Dengue patients are usually supportively-treated until they recover without any specific treatment measures. Several studies have shown that the level of viremia correlates with the severity of disease with high viremia often seen in severe dengue. Hence, antivirals that can reduce the level of viremia or the viremic phase could possibly reduce the severity of dengue.

Plants and plant's derived compounds remain an important source for the discovery and the development of new antiviral drugs because of their expected low side effects and their high accessibility in the nature [2-4]. There have been numerous reports on the antiviral activity of various phytochemicals against dengue viruses and these include various flavonoids [5-8]. Flavonoids are basically low molecular weight phenolic compounds found widely in different kinds of plants. Different types of flavonoids can be found in fruits, roots, nuts, seeds, bark, steams and flowers of plants. These include quercetin which can be found in some foods and fruits such as green and black tea, apple, onion, citrus, tomato and some other plants [9,10]. Antiviral activities of various other flavonoids have also been reported against some viruses including human cytomegalovirus (HCMV), HSV-1, HSV-2 and some types of human adenoviruses [11-13].

In the present study, we are interested to examine the anti-dengue virus properties of quercetin, hesperetin, naringin and daidzein. Hesperetin is a flavonone and its glycoside form, hesperidin is water soluble and it could be found in various citrus fruits. After ingestion of hesperidin, its sugar moiety is released from the backbone of this compound and the aglycone form known as hesperetin can be released. *In vitro *antiviral activities of hesperetin have been reported against some RNA viruses [14-16]. Naringin on the other hand, is a flavonone glycoside found abundantly in grapefruit juice. Antiviral activity of naringin were reported against HSV-1 and HSV-2 but this finding remains controversial [12,17]. Daidzein is an isoflavone found in soybeans and its antiviral activity against influenza viruses has been reported [18]. Currently, there is no published data on the possible anti-dengue virus activities of quercetin, hesperetin, naringin and daidzein. Therefore, in this study we evaluated these compounds activities on DENV-2 (NGC strain) replication in cell culture system. The effects of each compound were evaluated against different stages of dengue virus replication including virus adsorption, intracellular replication and direct virucidal activities.

## Methods

### Bioflavonoids

Four different types of bioflavonoid, quercetin, naringin, hesperetin (Sigma-Aldrich, St. Louis, MO, USA) and daidzein (Indofine Chemical Co. Inc., Hillsborough, NJ, USA) were evaluated for their potential activities against dengue virus replication. Dimethyl sulfoxide (DMSO) (Sigma-Aldrich, St. Louis, MO, USA) was used to dissolve the lyophilized form of compounds and prepared stock solutions (20 mg mL^-1^) were stored at -20 C. Stock solution was diluted using cell culture medium and sterilized by a syringe filter with 0.2 μm pore size (Millipore, MA, USA) right before each experiment.

### Cells and virus

C6/36 mosquito cell line derived from *Aedes albopictus *and Vero (African green monkey kidney) cell line were used in this study. Both cell lines were maintained and propagated in Eagle's Minimum Essential Medium (EMEM) (Gibco, NY, USA) containing 10% fetal bovine serum (Gibco, NY, USA). Cultured C6/36 and Vero cells were incubated at 28 C and 37 C, respectively in 5% CO_2 _humidified chamber. At the time of virus propagation, serum concentration was reduced to 2%. Dengue virus type-2 (DENV-2) New Guinea C strain (NGC) was propagated using C6/36 cell line and harvested after CPE presentation on day seven post-infection. After titration, viral stock was stored at -70 C. Cell lines and virus were provided by Virology laboratory of the Tropical Infectious Disease Research and Education Center, Faculty of Medicine, University of Malaya (Kuala Lumpur, Malaysia).

### *In vitro *cytotoxicity assay

MTT assay was performed following the manufacturer's instructions (Promega, WI, USA). Briefly, confluent Vero cells in 96-well cell culture microplates were treated with different concentrations of each compound in triplicate. The treated cells were incubated for four days at 37 C followed by the addition of 15 μl of MTT solution to each well. The microplate was incubated at 37 C for 4 h. Then, 100 μl of the solubilisation/stopping solution was added to each well. The optical density (OD) of wells was measured at 570 using 96-well plate reader (TECAN, Mannendorf, Switzerland). Dose-response curves were plotted using Graph Pad Prism 5 (Graph Pad Software Inc., San Diego, CA).

### Pretreatment of cells with bioflavonoids

In order to determine the prophylactic anti-dengue activity of compounds, different concentrations of compounds were added to the Vero monolayer cells in triplicate at different times, 5 h before virus infection. After 5 h of pre-infection treatment, the cells were washed twice with sterile PBS and then 200 FFU of DENV-2 was inoculated to the cells and incubated at 37 C for 1 h. To determine the effects of continuous treatment, different concentrations of each compound were added to the Vero cells, 5 h pre-infection and continuously for 4 days post-infection.

In a separate experiment, antiviral activity of compounds against intracellular replication of DENV-2 was performed by inoculation of 200 FFU of virus to each well in triplicate. After adsorption of virus to the cells for 1 h at 37 C, the cells were washed with PBS to eliminate the unabsorbed viruses. Then, different concentrations of each compound were added to the cells, followed by 4 days of incubation at 37 C. DENV RNA was then quantified using quantitative RT-PCR. In another experiment, Vero cells at 80% confluency were infected with 200 FFU of DENV-2 in the presence or absence of different concentrations of each compound. The microplate was kept at 37 C for 1 h for virus adsorption. Then the cells were washed two times by sterile PBS and incubated at 37 C for four days.

Direct virucidal effects of the bioflavonoids were investigated by incubating DENV-2 suspension containing 200 FFU with an equal volume of the different concentrations of each compound for 2 h at 37 C. Then, Vero cells were infected with the treated viral suspension in triplicate. After 1 h adsorption at 37 C, cells were washed twice with PBS in order to remove unabsorbed viruses. Then the microplate was incubated at 37 C for 4 days.

### Antiviral activity assay

Antiviral activities of the tested compounds were evaluated by measuring the reduction in number of viral foci. Briefly, confluent monolayers of Vero cells were prepared in 24 wells cell culture microplate. Then the infected cells treated using different procedures described above were overlaid with 1.5% of Carboxy Methyl Cellulose (CMC) (Sigma-Aldrich, St. Louis, MO, USA) containing EMEM. Viral foci were visualized using peroxidase-based foci staining assay four days post infection [19]. The numbers of DENV-2 foci were counted using a stereomicroscope and the titer of virus was expressed as Foci-Forming-Unit (FFU). The percentage of viral foci reduction (RF %) compared with controls was calculated as follows: RF (%) = (C-T) × 100/C. Where, C is the mean of the number of foci for negative control well (without compound) and T is the mean of the number of foci in treated wells. Reduction in the number of viral foci was further verified using quantitative RT-PCR (qRT-PCR).

### Dengue virus quantitative RT-PCR (qRT-PCR)

Quantitative RT-PCR was performed to determine the effects of bioflavonoids on DENV replication by quantifying DENV-2 genomic RNA copies based on a method described previously with some modifications [20]. Briefly, intracellular and extracellular DENV-2 RNAs were harvested from the DENV-infected Vero cells. Viral RNA was extracted using two types of RNA extraction kits (QIAamp Viral RNA mini kit and RNeasy mini kit) (Qiagen, Hilden, Germany). Quantitative RT-PCR assay was performed by adding 1 μl of extracted DENV RNA to the SensiMix SYBR green reagent (Quantace, Watford, United Kingdom) which contained 7.4 μl ddH2O, 10 μl 2X SensiMix One-Step, 0.4 μl 50X SYBR Green solution, 10 units of RNAse Inhibitor, 50 pmol of forward (DNF) and also reverse (D2R) primers [21]. All samples were assayed in triplicate. The amplifications were performed using the DNA Engine Opticon system (MJ Research/Bio-Rad, Hercules, CA) with the following thermal conditions: reverse transcription at 50°C for 30 min, initial denaturation at 95°C for 10 min, followed by 45 cycles of 95°C for 15 sec, 59°C for 30 sec and 72°C for 30 sec. Melting curve analysis was subsequently performed at temperature from 60°C to 98°C to verify the assay specificity. For absolute quantitation of the viral RNA, a standard curve was established with a serially diluted RNA extracted from DENV-2 stock with known titer.

### Statistical analysis

Graph Pad Prism for Windows, version 5 (Graph Pad Software Inc., San Diego, CA, 2005) was used to determine the cytotoxic concentration 50 (CC_50_) and inhibitory concentration 50 (IC_50_) values of bioflavonoids. Selectivity Index value (SI) was determined as the ratio of CC_50 _to IC_50 _for each compound.

## Results

### Cytotoxicity of bioflavonoids

**Figure 1:**
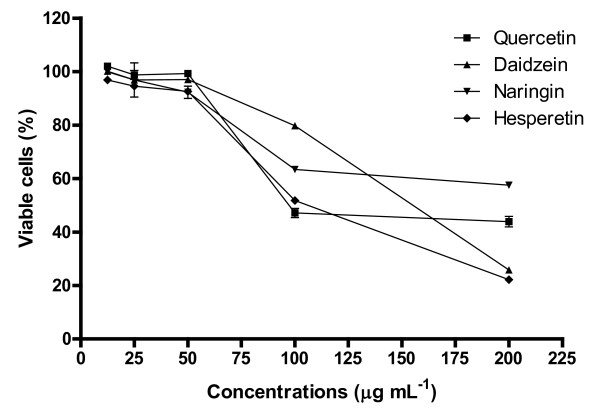
**Cytotoxicity of flavonoids against Vero cells**. MTT assay was performed on Vero cells after 96 h treatment with increasing concentrations of the flavonoids. Results are presented as percentage of cell viability from triplicate assays.

MTT assay was used to determine cytotoxicity of each bioflavonoid on Vero cells and the CC_50 _value of each compound was calculated (Table 1 and Figure 1). Vero cells were treated by bioflavonoids for 4 days which was the same duration used for antiviral activity assay. Results showed that hesperetin with CC_50 _= 110.3 ± 0.32 μg mL^-1 ^is the most cytotoxic compound for Vero cells compared to the other tested compounds. Quercetin and daidzein showed lower toxicity against Vero cells at CC_50 _252.6 ± 0.17 and 147.8 ± 0.31 μg mL^-1^, respectively. Meanwhile, naringin with CC_50 _= 230.3 ± 0.19 μg mL^-1 ^showed the least cytotoxic effects against Vero cells. Cells treated with vehicle control, 1% DMSO did not show any cytotoxicity against Vero cells.

**Table 1:** Cytotoxicity assays were performed using the MTT assay method described in text

Flavonoids	CC_50_(μg mL^-1^)
Quercetin	252.6 ± 0.17

Daidzein	147.8 ± 0.31

Naringin	230.3 ± 0.19

Hesperetin	110.3 ± 0.32

The CC_50 _of bioflavonoids were calculated using Graph Pad Prism Version 5 (Graph Pad Software Inc., San Diego, CA.). Results are presented as mean ± SD of triplicate experiment.

### Antiviral activity of bioflavonoids

**Figure 2:**
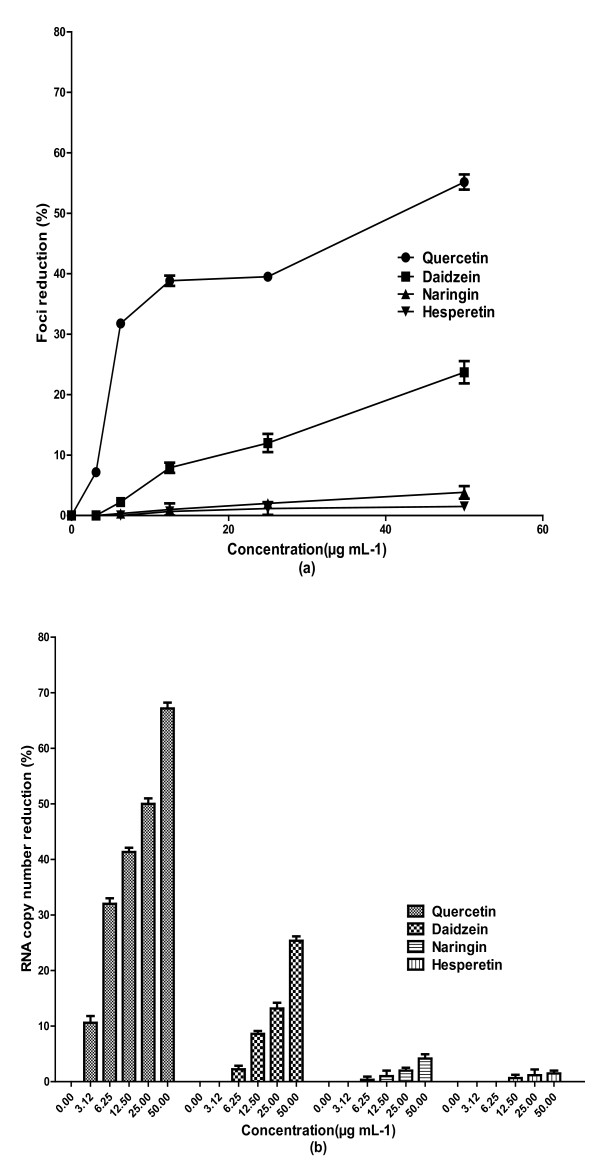
**Anti-viral assay of flavonoids against DENV-2 intracellular replication**. Foci forming unit reduction assay (FFURA) was used to evaluate the *in vitro *anti-dengue virus activities of flavonoids after viral adsorption (**a**) and the respective DENV-2 RNA copies were quantified using qRT-PCR (**b**). The percentages of foci reduction (% RF) were calculated relative to the untreated controls maintained in parallel. Data from triplicate assays were plotted using Graph Pad Prism Version 5 (Graph Pad Software Inc., San Diego, CA. USA).

Results of Vero cells pre-treatment with the compounds showed that 50 μg mL^-1 ^of quercetin could decrease the number of DENV-2 foci by 14% ± 1.5 when compared to the non-treated cells. However, there was no prophylactic activity against DENV-2 from other compounds (data not shown). In post-adsorption assay, quercetin exhibited the most significant antiviral activity against DENV-2 amongst the bioflavonoids tested with IC_50 _= 35.7 μg mL^-1 ^(Figure 2a). The SI value for quercetin in post-adsorption assay was relatively high at 7.07. It was also demonstrated that the level of DENV-2 specific RNA production in the presence of 50 μg mL^-1 ^of quercetin decreased by more than 67% ± 1 when compared to the non-treated infected cells (Figure 2b). Daidzein showed very weak anti-dengue activity with IC_50 _= 142.6 μg mL^-1 ^when the infected cells were treated after DENV-2 adsorption (Figure 2a). Its related SI value was 1.03. The levels of DENV-2 RNA production in the presence of 50 μg mL^-1 ^of daidzein decreased by only 25.3% ± 0.7 when compared to the non-treated infected cells (Figure 2b). Naringin and hesperetin did not exhibit any anti-dengue activity when they were used after adsorption of DENV-2 to the Vero cells (Figure 2).

**Figure 3:**
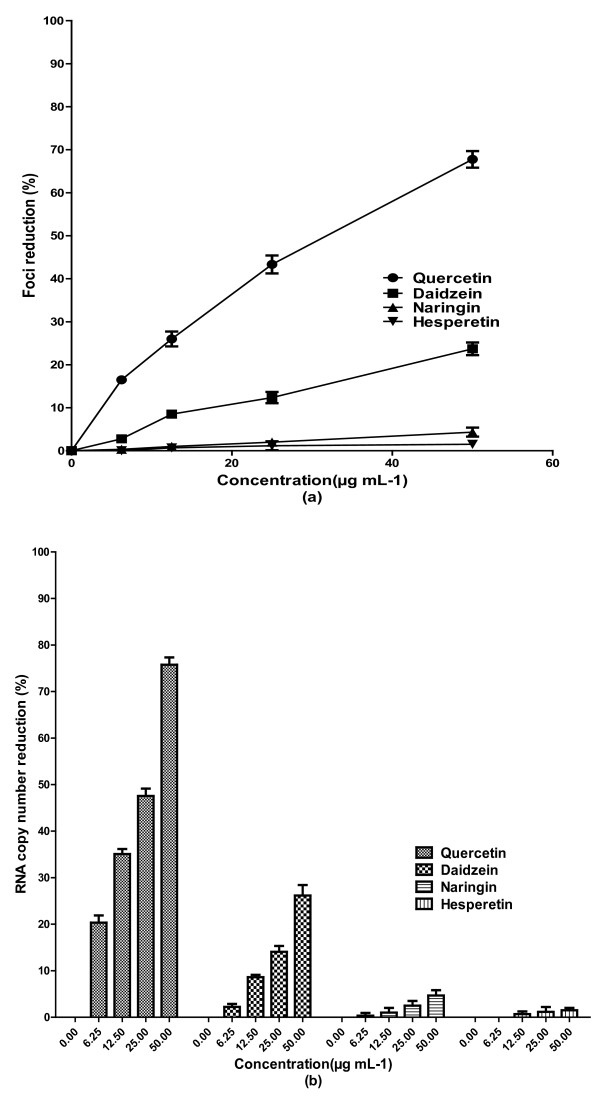
**Anti-viral effects of continuous treatment with flavonoids against DENV-2 replication**. Foci forming unit reduction assay (FFURA) was used to evaluate the *in vitro *anti-dengue virus activities of the flavonoids. Cells were treated with the flavonoids at 5 h before infection and continuously treated with fresh flavonoids upto 4 days post infection (**a**). The respective DENV-2 RNA copy numbers were quantified using qRT-PCR (**b**). The percentages of foci reduction (% RF) were obtained by comparing against untreated controls maintained in parallel. Data from triplicate experiments were plotted using Graph Pad Prism Version 5 (Graph Pad Software Inc., San Diego, CA).

**Figure 4:**
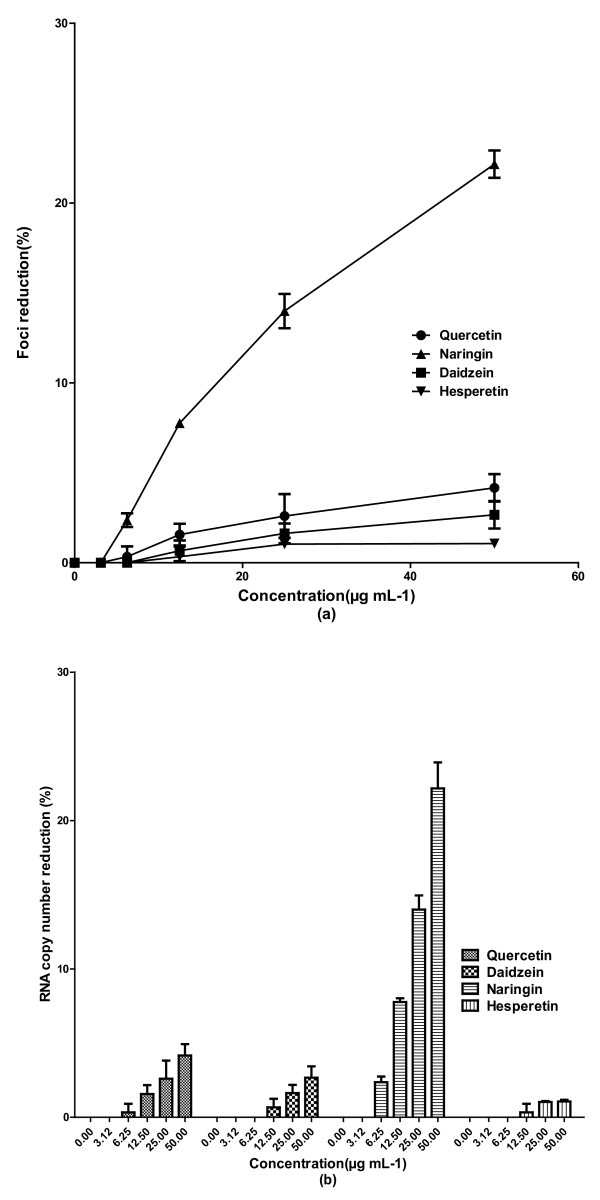
**Anti-adsorption activity of flavonoids against DENV-2**. Flavonoids were added directly to virus inocula for 2 h at 37 C. The inocula were used to infect Vero cell monolayers in 24 wells cell culture microplates. The reduction in foci forming unit was calculated relative to the controls maintained in parallel (**a**) and the respective DENV-2 RNA copy numbers were quantified using qRT-PCR (**b**). Data from triplicate experiments were plotted using Graph Pad Prism Version 5 (Graph Pad Software Inc., San Diego, CA).

Although there was no significant direct virucidal activity against DENV-2 by quercetin, continuous treatment of cells from 5 h before virus infection up to 4 days post-infection exhibited anti-dengue activity with IC_50 _= 28.9 μg mL^-1 ^(Figure 3a). The SI value for continuous treatment with quercetin was 8.74 and higher than the SI value (7.07) for post-adsorption assay. In addition, the level DENV-2 RNA production decreased by more than 75.7% ± 1.57 when Vero cells were treated with 50 μg mL^-1 ^of quercetin, 5 h before virus infection and up to 4 days post infection (Figure 3b). There was no significant change in the antiviral activity of daidzein when cells were treated continuously from 5 h before virus infection up to 4 days post infection comparing to its anti-dengue activity for post-adsorption treatment (Figure 1). No significant antiviral activity for naringin and hesperetin was observed for the continuous treatment against DENV-2 (Figure 2). However, naringin exhibited anti-adsorption activity when it was added to the cells at the same time of virus adsorption. The IC_50 _value for naringin was 168.2 μg mL^-1 ^and its related SI was 1.3 (Figure 3a). There was a reduction of 25.8% ± 0.76 in DENV-2 RNA production in the presence of 50 μg mL^-1 ^of naringin (Figure 4a).

**Figure 5:**
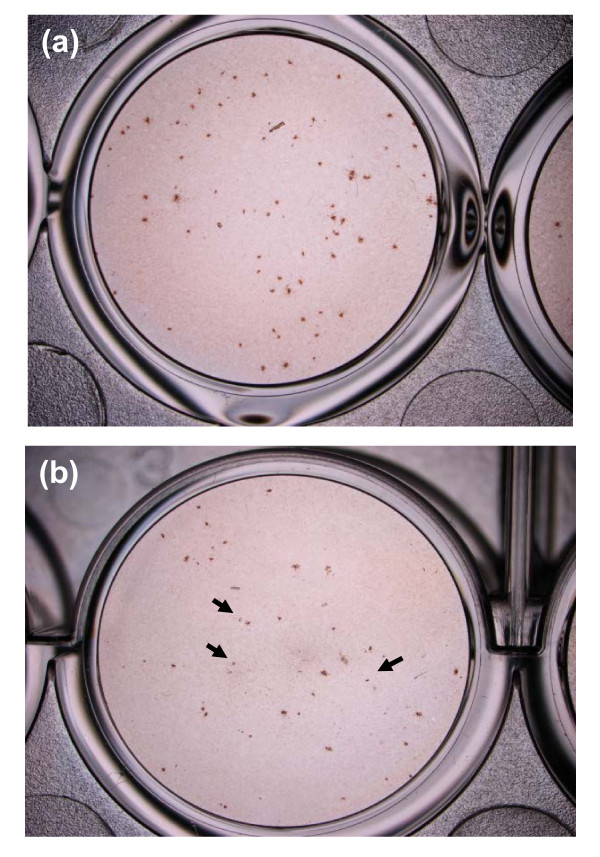
**Foci size reduction in DENV-infected cell treated with quercetin**. Foci forming unit reduction assay (FFURA) was used to evaluate the in vitro anti-dengue virus activity of quercetin after viral adsorption. Foci of the non-treated infected cells (**a**) compared with foci in cells treated with quercetin (50 μg/ml) postadsorption (**b**). Arrows indicate foci which are more diffused, less intensely stained and smaller than the foci in non-treated cells.

The majority of the viral foci in cells treated with 50 μg/ml quercetin appeared smaller, less intensely stained and more diffused within the focus (Figure 5b), compared to the larger, well-defined and more intensely stained foci of the untreated cells (Figure 5a). This observation is consistent with the reduction of the percentage of foci and RNA copy number.

Results from the direct virucidal activity evaluation of each compound showed that there was no extracellular inhibitory activity against DENV-2 for all the tested compounds. Similarly, qRT-PCR analysis showed that there was no significant decrease in copy number of DENV-2 RNA following direct treatment of DENV-2 with the different concentrations of each compound (data not shown).

## Discussion

Recent studies have shown that flavonoids such as glabranine and 7-O-Methyle glabranine exhibited significant antiviral activities against dengue virus. *In vitro *treatment of infected cells with these flavonoids resulted in the reduction of intracellular replication of dengue virus by 76.9% and 75% following treatment with 25 μM of glabranine and 7-O-Methyle glabranine, respectively [22]. Similarly, other synthetic flavonoid derivatives also showed antiviral activity in HepG2 cells [23]. Whereas, pinostrobin was reported to inhibit DENV-2 NS2B/NS3 protease an enzyme important in dengue virus replication in an *in vitro *study [24]. These suggest that flavonoids as a group could consist of select compounds that possesses inhibitory activities against DENV. To investigate which of the many flavonoids could affect DENV infection, in the present study, we examined the potential effects of quercetin, naringin, hesperetin and daidzein on dengue virus infection of Vero cells. Unlike the previous studies which evaluated antiviral activity of flavonoids only after adsorption of virus to the cells [22,23], the present study evaluated antiviral activity by different treatment procedures tailored to determine prophylactic, post adsorption, continuous treatment and direct virucidal activities of quercetin, naringin, daidzein and hesperetin.

Our findings demonstrated that quercetin was the only compound among all tested flavonoids that consistently showed significant antiviral activity against DENV-2 in Vero cells. Selectivity indices for quercetin when the infected cells were treated or when uninfected cells were treated continuously 5 h before infection until 4 days post-infection were 7.07 and 8.74, respectively. The noted differences between SI values for quercetin could be due to the intracellular accumulation of quercetin during continuous treatment. A weak effect for prophylactic activity of quercetin however, was also observed. These findings suggest that the main anti-dengue activity of quercetin is likely due to its activity against the different stages of intracellular replication of DENV-2 instead of early stages of its replication cycle such as virus attachment or entry. Although, no direct virucidal activity or anti-attachment activity of quercetin was observed in the present study, antiviral activity of quercetin against human cytomegalovirus was reported with IC_50 _= 3.2 μM [13]. Quercetin was also reported to be effective against herpes viruses where it is more specific against HSV-1 with SI = 22 compared to HSV-2 with SI = 5.7 [11].

The mechanisms of how quercetin exerts its antiviral effects are not known. However, the effects of other flavonoids against cellular RNA polymerases and formation of the complex with RNA have been reported suggesting that quercetin could also affect the similar replication enzymes [25,26]. Sylimarin, a flavonoid found effective against hepatitis C virus (HCV), another member of flaviviridae family [27], inhibits virus replication by inhibiting the activity of viral RNA polymerase. In our study, results from the qRT-PCR supported the findings from the viral foci reduction assays that quercetin inhibits DENV-2 replication and the significant reduction in the DENV specific RNA suggests that quercetin may also target the virus replication machinery, namely by inhibiting the RNA polymerase.

Antiviral activity of naringin has been evaluated against few herpesviruses and rotavirus but their reported antiviral activities against HSV-1 and HSV-2 are inconclusive [12,17]. In addition it was reported that naringin did not exhibit any antiviral activity against another RNA virus, sindbis virus [14]. In the present study, the only anti-dengue activity of this flavonoid was demonstrated against adsorption and attachment of virus to the Vero cells and based on its antiviral activity (IC_50 _= 168.2 μg mL^-1^) and its related selectivity index (SI = 1.3), it may not be a good candidate for further development as anti-dengue drug. Similarly, daidzein activity against DENV-2 was not significant compared to quercetin (SI = 1.03). Continuous treatment of the infected Vero cells from 5 h before virus infection up to 4 days post infection did not improve its anti-dengue activity significantly. This compound therefore, could not be a suitable candidate for further development as anti-dengue drug. Hesperetin, the other flavonoid evaluated in our study, did not show no anti-dengue activity in any stages of virus infection and replication processes and this is despite the previously reported antiviral activity of hesperetin against sindbis virus [14]. Therefore, hesperetin is also not recommended for further investigations for anti-dengue drug development. In all our experiments, we showed that 0.5% of DMSO, the highest concentration of solvent used in the bioflavonoid treatment did not exhibit any antiviral activity against DENV-2 and this eliminated any probable antiviral activity from DMSO.

Findings from our study, suggest that there are select flavonoids including quercetin and fisetin, which are both flavonol, that exhibited significant DENV replication inhibition properties [28]. While the flavonoids in general share common basic molecular base structure, flavone (2-phenyl-1,4-benzopyrone), we showed here that the flavanone, hesperetin, and flavanone glycoside, naringin, showed no significant anti-DENV replication activities. In addition, we had earlier shown that naringenin [28], another flavanone metabolized from naringin and here, daidzein, an isoflavone also had no significant DENV replication inhibition properties. While quercetin was shown here to be effective in inhibiting DENV replication, its glycoside form, rutin (quercetin-3-O-rutinoside) showed no significant inhibition properties [28]. These suggest that while flavonol could be the basic molecule that possesses anti DENV replication properties, specific structural properties of the different flavonol derivatives would have different effects on the efficacy of the compounds against dengue.

The demonstration in vitro that flavonols including quercetin and fisetin possess anti DENV replication properties does not necessarily translate into immediate use of these compounds as antivirals against DENV. Further studies will be needed to demonstrate the antiviral activities of these compounds against different genotypes of dengue virus and in appropriate animal model. There is also a need to address the issue of the low bioavailability of quercetin especially for therapeutic use [29-31]. Several strategies to increase the bioavailability of quercetin that include using lipids and emulsifiers, co-crystalization of quercetin or using ester-based precursors have been investigated [32-34]. The other topic of research would be combination drug study. At its current calculated IC_50 _values, the antiviral efficacy of quercetin can be further improved possibly by combining it with other potential anti-dengue compounds. This is exemplified in a study that reported the synergistic effect of α- glucoside in combination with a standard antiviral drug, ribavirin is effective against dengue infection [35].

## Conclusions

In conclusion, the present study demonstrates that the bioflavonoid quercetin exhibited significant anti DENV replication properties. We further showed that quercetin affects intracellular DENV virus replication but not the DENV attachment and entry processes. These results together with the earlier findings reporting the anti DENV properties of fisetin, suggest that these flavonols could be further investigated for their specific uses as candidate anti-DENV therapeutics.

## Competing interests

The authors declare that they have no competing interests.

## Authors' contributions

KZ designed and carried out the antiviral and cytotoxicity studies and drafted the manuscript. BTT carried out the virus propagation and antiviral studies. SSS participated in the quantitative RT-PCR. WPF participated in the design of the study, performed statistical analyses and edited the manuscript. MRM participated in study design and provided all bioflavonoids. SAB conceived the whole study and edited the manuscript. All authors read and approved the final manuscript.
